# Nitrogen Acquisition by Invasive Plants: Species Preferential N Uptake Matching with Soil N Dynamics Contribute to Its Fitness and Domination

**DOI:** 10.3390/plants14050748

**Published:** 2025-03-01

**Authors:** Xingang Chang, Wenying Wang, Huakun Zhou

**Affiliations:** 1 School of Life Sciences, Qinghai Normal University, Xining 810008, China; 15935143264@163.com; 2 Northwest Institute of Plateau Biology, Chinese Academy of Sciences, Xining 810008, China; hkzhou@nwipb.cas.cn

**Keywords:** invasive plant, nitrogen preference, nitrate, ammonium, PSF, N transformation

## Abstract

Plant invasions play a significant role in global environmental change. Traditionally, it was believed that invasive plants absorb and utilize nitrogen (N) more efficiently than native plants by adjusting their preferred N forms in accordance with the dominant N forms present in the soil. More recently, invasive plants are now understood to optimize their N acquisition by directly mediating soil N transformations. This review highlights how exotic species optimize their nitrogen acquisition by influencing soil nitrogen dynamics based on their preferred nitrogen forms, and the various mechanisms, including biological nitrification inhibitor (BNI) release, pH alterations, and changes in nutrient stoichiometry (carbon to nitrogen ratio), that regulate the soil nitrogen dynamics of exotic plants. Generally, invasive plants accelerate soil gross nitrogen transformations to maintain a high supply of NH_4_^+^ and NO_3_^−^ in nitrogen-rich ecosystems irrespective of their preference. However, they tend to minimize nitrogen losses to enhance nitrogen availability in nitrogen-poor ecosystems, where, in such situations, plants with different nitrogen preferences usually affect different nitrogen transformation processes. Therefore, a comprehensive understanding requires more situ data on the interactions between invasive plant species’ preferential N form uptake and the characteristics of soil N transformations. Understanding the combination of these processes is essential to elucidate how exotic plants optimize nitrogen use efficiency (NUE) and minimize nitrogen losses through denitrification, leaching, or runoff, which are considered critical for the success of invasive plant species. This review also highlights some of the most recent discoveries in the responses of invasive plants to the different forms and amounts of N and how plants affect soil N transformations to optimize their N acquisition, emphasizing the significance of how plant–soil interactions potentially influence soil N dynamics.

## 1. Introduction

Plant invasions play a significant role in global environmental change [1], impacting community structure [2] and altering ecosystem processes and functions [3]. The changes in native species [4,5], plant biomass [4,6], water conditions, nutrient cycling [6], and fire frequency [5] are often associated with the spread of invasive plants. Soil resource acquisition, whether through the depletion or enhancement of resource supply [7], along with the disturbance of microbial-mediated resource supply [8], are considered critical drivers of plant invasion, leading to extensive resource limitations for native plants. The growth of native species is typically inhibited by the superior competitive ability of invasive plants for soil nutrient uptake [9]. For instance, nitrogen is a crucial element required for the biosynthesis of key cellular components. Invasive plants absorb and utilize nitrogen more efficiently than native plants [10,11] through enhanced root development and altered root morphology [12]. Furthermore, increased nutrient-rich litter inputs may alter soil nitrogen transformation by directly affecting soil pH and supporting specific functional groups [13]. These changes in resource flows [14,15] create feedback mechanisms that facilitate the successful invasion of native plant communities, ultimately impacting biodiversity and ecosystem function.

Nutrient acquisition strategies, particularly species’ preferences for specific chemical forms of nutrients, influence the coexistence and distribution of species [16] and may facilitate the successful invasion of plants [17]. One significant reason that plant species exhibit preferences for different nitrogen (N) forms is as an adaptive strategy to N limitation. Dominant species tend to exploit the most readily available forms of N, while less productive species utilize the least available N forms [18]. However, some invasive plants demonstrate broader soil N niches, allowing them to assimilate various forms of available N or to exhibit stronger niche adjustments to changes in preferred N forms during competition [19]. To acquire more nutrients and reduce losses of mineral forms of N in the ecosystem, invasive plants tend to access different forms of N supply at varying soil depths and times [20]. Nevertheless, there is limited evidence that N-fixing invasive plants prefer specific N forms. Instead, they tend to enhance N fixation as a different mechanism to accelerate N fluxes and increase N pools [21].

Plant nitrogen (N) preference is often linked to the availability of specific N forms in the soil, which are interrelated with the predominant soil N transformations [15]. The abundance and forms of accessible N in soils are closely associated with the processes of depolymerization and the mineralization of soil organic N to ammonium, followed by its oxidation to nitrate [22]. Among these processes, nitrification can significantly increase N mobility and the potential loss from the soil system, ultimately affecting nitrogen supply for plants. Plants benefit more when the preferred N form aligns with the dominant inorganic N form and N transformations in the soil. For instance, ammonium (NH_4_^+^) is the preferred inorganic N form taken up by plants in acidic soils, characterized by a low nitrification/mineralization ratio in the soil [23]. In contrast, in neutral and alkaline soils, plants primarily utilize nitrate (NO_3_^−^) as the dominant inorganic N form, which corresponds to a high nitrification/mineralization ratio in the soil [24].

The preference of plants for different N forms uptake is expected to influence soil N transformations to enhance their N acquisition. Although this hypothesis has yet to be fully elucidated, several experimental studies have provided support for it. Plants that prefer ammonium, such as sugarcane and tea, exhibit a higher uptake of NH_4_^+^ compared to microbial NH_4_^+^ immobilization rates. Additionally, microbes uptake more NO_3_^−^ with increased gross rates of heterotrophic nitrification [25]. Optimized N transformations were also observed in plants with a preference for NO_3_^−^. It was shown that organic N oxidation to NO_3_^−^ (ONrec) is enhanced, contributing significantly to NO_3_^−^ production when NH_4_^+^ oxidation (O NH_4_^+^, the autotrophic pathway) is insufficient to meet the plants’ NO_3_^−^ demands [26]. This phenomenon explains why invasive plants, such as *Andropogon yunnanensis*, *E. adenophora*, *F. bidentis*, and *Wedelia trilobata*, which have a preference for NH_4_^+^-N, are often found in areas where the predominant form of N is NO_3_^−^-N [27,28,29,30].

Most studies have primarily focused on how plant nitrogen (N) preference influences plant invasion through non-competitive designs or under competition, without accounting for soil N transformation characteristics. Furthermore, the existing literature on the impact of exotic invasions on soil N transformation has largely concentrated on how microbial composition, functional capabilities, and physicochemical parameters changed following invasion. However, there is a notable gap in the literature regarding the comprehensive understanding of the interactions between invasive plant species’ preferential nitrogen (N) form uptake and the characteristics of soil N transformations. A thorough review is necessary to scientifically evaluate how exotic species optimize their nitrogen acquisition by influencing soil nitrogen dynamics based on their preferred nitrogen forms. This understanding is essential for elucidating how exotic plants enhance nitrogen use efficiency (NUE) and minimize nitrogen losses through denitrification, leaching, or runoff, which are critical for the success of invasive plant species. Addressing this gap will guide future research and provide a deeper understanding of the role of nitrogen in plant invasions. Filling this gap will significantly enhance our knowledge and aid in the formulation of effective management strategies.

## 2. Plant–Soil Feedback Influence Soil N Transformations of Invasive Plants

A common anticipation regarding plant–soil feedback (PSF) and invasion is that the expansion of invasive plants will occur when these exotic species possess a greater ‘home’ advantage and experience less negative PSF compared to native species. Plant–soil feedback consist of ‘effects’ and ‘responses’ [31]. Plant effects influence soil resources by altering the quantity and quality of litter and root exudates, while plant responses refer to traits (such as growth rate and nutrient use efficiency) that react to changes in soil resource quantities and qualities. A species exhibiting positive feedback performs better in its native soil than in the soil of other species [32]. Conversely, negative feedback occurs when the opposite is true [32]. Invasive species often escape the direct negative effects of natural enemies, such as host-specific soil pathogens, and thrive in their non-native soil environments [33]. Native plants, on the other hand, are more likely to accumulate pathogens, which further limits their growth in their home soil. Additionally, invasive plants tend to benefit more from interactions with mycorrhizal fungi and exhibit neutral or positive feedback [34]. However, another study indicated that the response of exotic species to soil-conditioning effects is variable and not consistently observed [32].

Soil community structures and functions mediated by plant invasion primarily depend on two distinct biotic loops: the litter-based loop and the living root-based loop. Invasive plants produce more litter with higher quality (lower C:N ratio), which accelerates nutrient cycling and provides greater availability of nutrients for plants [35]. The living root-based loop mainly alters the soil microbiota by secreting root exudates, which supply carbon and nitrogen substrates, such as amino acids, sugars, and carboxylic acids, for microbial growth [36]. Additionally, root exudate compounds have a more extensive effect by acting as signaling molecules, repellents, stimulants, inhibitors, or attractants [37]. For example, the exudation of root compounds from plant roots selectively recruits soil microbiota, potentially influencing the growth and herbivore defense of subsequent generations [38]. Although it has been widely hypothesized that plant–soil feedback (PSF) play an important role in exotic species invasion, the initial abundance of the exotic species [32] and the equivalence of dispersal and life history characteristics also need to be considered, as the establishment of invaders often precedes invader-mediated PSF.

Exotic species optimize their nitrogen acquisition by influencing soil nitrogen transformations and enhancing microbial activity and biomass. The productivity of invasive plants is likely much greater than that of native plants, which may contribute to increased soil nitrogen pools. For instance, *Spartina alterniflora* exhibits higher net primary production, providing it with a significant nutrient absorption advantage [39,40]. Alternatively, increased litter input, which adds labile nitrogen and labile carbon to the soil, promotes microbial activity and nitrogen transformations [41,42]. Exotic plants characterized by lower leaf and fine root carbon-to-nitrogen (C:N) ratios alter the carbon and nitrogen cycles of the soil through higher decomposition rates and increased nutrient immobilization, often associated with elevated rhizosphere soil and microbial biomass carbon and nitrogen concentrations [43]. Invasive plants also negatively impact the biomass and abundance of soil biota; the roots of these plants secrete allelopathic compounds that suppress bacterial biomass, which can enhance root growth and nutrient uptake by reducing nutrient leakage [35].

## 3. Invasive Plants Modify the Soil N Cycle According to the Plant Species N Preference

### 3.1. Ammonium Uptake Preference

**Figure 1:**
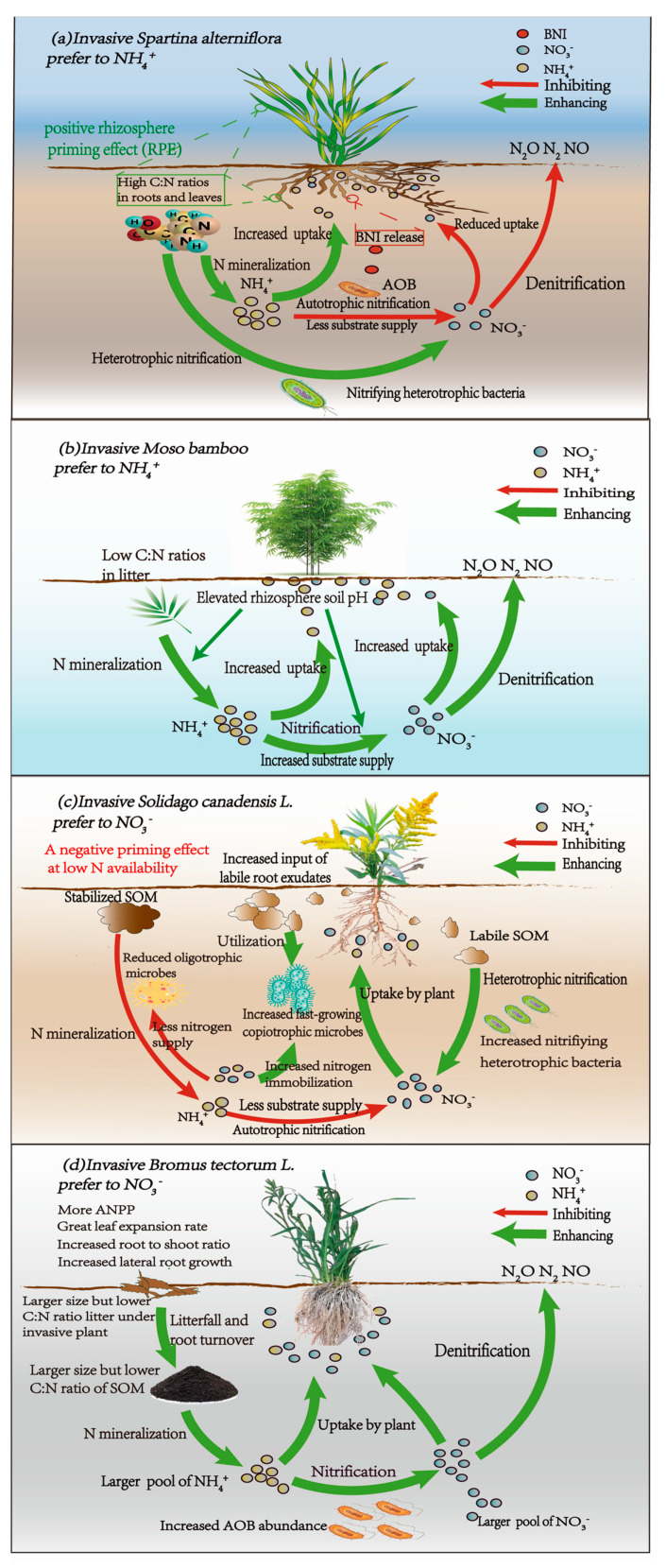
A schematic diagram illustrating soil N transformation among various types of invasive plants with different chemical N preferences. The color of the lines among the N pools represents the positive (green) and negative (red) effect of invasive plants on each process.

The manner in which invasive plants modify the soil nitrogen (N) cycle may depend on the availability of nitrogen. Exotic species that utilize nitrogen rapidly tend to thrive in nitrogen-rich ecosystems, while those that can accelerate soil nitrogen transformations are more likely to invade nitrogen-poor ecosystems. Most invasive plants that prefer NH_4_^+^ are typically associated with higher ammonification rates in soil, which may enhance nitrification rates through increased substrate concentration [19,44]. For instance, one study demonstrated that invasive bamboo *(Phyllostachys edulis)* shoots can grow at a rapid rate by enhancing ammonification and nitrification, which are considered the dominant driving forces for bamboo expansion into adjacent forests [45] (Figure 1b) (Table 1). Invasive plants with a preference for NH_4_^+^-N uptake in low nitrogen availability conditions tend to maintain a high NH_4_^+^ supply by enhancing ammonification and suppressing nitrification and denitrification. Soil nitrogen availability has been recognized as a limiting factor for plant growth in Australian and African savannas. Invasive *Andropogon gayanus* and several other species have developed adaptive strategies for conserving soil nitrogen by releasing biological nitrification inhibition (BNI) [46] (Table 1). Lower nitrification minimizes nitrogen (N) losses to the environment, resulting in relatively higher soil ammonium availability [29].

A recent study by Chen et al. [47] demonstrated that invasive *Spartina alterniflora*, which exhibits a preference for NH_4_^+^-N uptake, optimizes its nitrogen acquisition by accelerating soil gross nitrogen transformations (Table 1). This includes increased nitrogen mineralization, decreased ONH_4_ (the oxidation of NH_4_^+^ to NO_3_^−^), and enhanced ONrec (the oxidation of recalcitrant organic nitrogen to NO_3_^−^) (Figure 1a). The observed increase in nitrogen mineralization is likely due to a positive rhizosphere priming effect (RPE) [48], which may be associated with the enhanced release of organic substrates, such as organic acids and low-molecular-weight soluble sugars, as well as promoted microbial activity [49]. The lower ONH_4_, previously considered the primary pathway for converting NH_4_^+^ to NO_3_^−^, is linked to reduced abundances of ammonia-oxidizing bacteria (AOB) as well as the influence of biological nitrification inhibition (BNI) [50] (Figure 1a). Some plants may selectively recruit heterotrophic microbes by secreting exudates with higher C:N ratios, which can outcompete nitrifying bacteria and reduce nitrification [51] (Figure 1a). Although earlier studies suggested that NH_4_^+^-preferring plants might enhance ONrec [25,52] by collaborating with nitrifying heterotrophic bacteria and fungi [53], this area has received limited investigation [54]. An increase in ONrec can improve NO_3_^−^ availability in response to restricted ONH_4_ under conditions of higher total organic carbon (TOC) and TOC/TN ratios [39]. Acetic acid is regarded as a signaling molecule that mediates fungal activity and triggers the ONrec process [55].

**Table 1:** The soil N dynamics among various types of invasive plant with different chemical N preferences. We preferentially consider the studies using the ^15^N tracing tool with an advantage that considers both soil N transformations and plant N uptake in terrestrial ecosystems.

Preference for Different Chemical N	Soil Chemical Properties and N Dynamics	Invasive Plants	Reference
A radical preference to ammonium	Increased ammonium and nitrate pool sizes, enhanced net ammonification, enhanced net nitrification and denitrification, increased abundance of *AOA-amoA, nifH, nirS, and nosZ*	*Moso bamboo (Phyllostachys edulis)*	[45]
		*Cenchrus spinifex*	[56]
		*Paspalum notatum*	[19]
		*soft brome (Bromus hordeaceus* L.)	[44]
		*Chromolaena odorata*	[27]
A conservative preference to ammonium	Increased ammonium supply, lowered nitrate concentration, promoted N mineralization, lower nitrification and denitrification reduce losses of nitrogen (N) leading to greater N availability, lowered abundance of *AOB*, invaded low N soils successfully	*Stellera chamaejasme* L.	[57]
		*Centaurea stoebe* L.	[58]
		*Spartina alterniflora*	[47]
			[59]
		*Andropogon gayanus*	[29]
		*Moso bamboo (Phyllostachys edulis)*	[60]
		*Aegilops triuncialis and Elymus caput-medusae*	[61]
A radical preference to nitrate	Enhanced nitrate/inorganic N ratios, higher litter decomposition and soil nitrate concentrations, increased mineralization and nitrification rates, higher gross rates of ammonium and nitrate consumption, increased abundance of ammonia-oxidizing bacteria	*Avena barbata*	[62]
		*Microstegium vimineum (stiltgrass)*	[63]
			[64]
		*Bromus tectorum, Centaurea stoebe and Euphorbia esula*	[65]
		*Bromus tectorum* L.	[66]
			[67]
A conservative preference to nitrate	Increased soil nitrate availability, a higher nitrate-N uptake rates than the ammonium-N uptake rates, increased soil net nitrification rate but decreased soil net mineralization, a greater portion of the mineralized N was nitrified, invaded low N soils successfully	*Ligustrum sinense Lour*	[68]
		*Mikania micrantha*	[69]
		*Solidago canadensis* L.	[70]
		*Pteridium aquilinum*	[71]

Alien plants can minimize losses of nitrate and nitrogen oxides by influencing the activity of denitrifying enzymes and the function of denitrifying bacteria in the soil. Invasive species such as big Asian knotweeds (*Reynoutria sachalinensis*) may promote nitrogen retention in the ecosystem by limiting both nitrification and denitrification [72]. This phenomenon may be triggered by (i) a reduced abundance of denitrifiers in the soil [72]; (ii) a decrease in denitrifying enzyme activity (DEA) due to the disruption of anaerobic conditions, which results from increased oxygen availability during *Fallopia* invasion; and (iii) a reduction in DEA stemming from limited nitrate availability in areas where nitrification activity is also suppressed.

### 3.2. Nitrate Uptake Preference

Some invasive plants utilize nitrate more effectively than ammonium due to the lower cost of assimilated nitrate [73]. Increased rates of nitrification provide more nitrate to exotic plants, thereby enhancing their fitness and dominant position within the community. Hawkes et al. [62] found that plant invasions impact nitrogen cycling, resulting in a higher abundance of soil nitrifying communities and elevated nitrification ratios with the emergence of exotic grasses, although mineralization rates did not show similar increases. A possible explanation for this phenomenon is that a greater proportion of ammonia derived from mineralization was nitrified. However, little is known about whether invaders are the cause or the consequence of these conditions. Lee et al. [63] conducted both field studies and garden experiments, collecting soils from invaded and uninvaded plots to estimate net nitrification potentials. Subsequently, native species were planted, and half of the plots were sown with invasive plant seeds. Net nitrification potentials were also assessed in the garden experiment. Lee’s findings revealed greater nitrate to inorganic nitrogen ratios and nitrification potentials in the soil of invasive plant under both natural and experimental conditions, indicating that plant invasion drives these changes in the nitrogen cycle [63].

Invasive plants exhibiting a preference for NO_3_^−^-N uptake demonstrate higher above-ground net primary productivity (ANPP) [65,74], increased allocation to root biomass [75], and elevated soil nitrate and nitrification rates [74] (Figure 1d). The influence of above-ground processes and plant traits may elucidate these observations. Different nitrogen forms can affect the competitive capacity of invasive plants by influencing specific plant traits. For instance, the expansion rate of leaves [76], maximum dry matter production [76], and lateral root growth [77] are greater when NO_3_^−^ is the nitrogen source compared to NH_4_^+^. A recent study [78] found that the root-to-shoot ratio of invasive species increased with higher soil NO_3_^−^ content, while it decreased with elevated soil NH_4_^+^ content. Consequently, nitrate uptake is crucial for plant development and significantly impacts the ANPP of invasive species. Increased ANPP leads to greater nitrogen inputs and soil nitrogen storage through enhanced litter accumulation and decomposition beneath invasive plants, which accelerates nitrification and subsequently provides a larger quantity of NO_3_^−^ for plant uptake. This dynamic ultimately creates a positive feedback loop that enhances the fitness of exotic grasses (Figure 1d).

How do exotic plants with a preference for NO_3_^−^-N uptake adapt to low nutrient environments? Given that NO_3_^−^ can easily escape from soil systems, this phenomenon may exacerbate nitrogen limitation in the soil. Research has indicated that the invasive plant *Solidago canadensis* L. (*S. canadensis*) (Table 1), which prefers NO_3_^−^-N and exhibits tolerance to low nutrient stress, significantly reduces the nitrogen mineralization rate and the autotrophic nitrification rate [79]. This reduction occurs due to a negative priming effect on the decomposition of original soil organic matter [49] (Figure 1c). Dijkstra [80] noted that a greater proportion of labile root exudates compared to recalcitrant soil organic matter can decrease the release and activity of extracellular enzymes, resulting in lower soil organic nitrogen mineralization rates [81,82]. Surprisingly, the heterotrophic nitrification rate was stimulated to meet the NO_3_^−^-N requirements of both plants and microbes [79]. In this context, the direct oxidation of organic nitrogen serves as a primary pathway for the production of NO_3_^−^-N, allowing plants to control the supply of NO_3_^−^-N in response to nitrogen demand by stimulating heterotrophic nitrifiers through root exudates (Figure 1c). This mechanism contributes to the higher environmental adaptability of *S. canadensis* in conditions of limited nitrogen supply.

## 4. Mechanisms That Regulate Exotic Plant–Soil N Transformation

It remains an open question what mechanisms regulate the transformation of nitrogen in the soil by exotic plants in response to their preference for different chemical forms of nitrogen. While there may be several potential pathways, we will specifically focus on three non-mutually exclusive mechanisms.

### 4.1. BNI Release

Compelling evidence has accumulated that supports the hypothesis that biological nitrification inhibition (BNI) capacity is likely an adaptive mechanism that allows plants to retain and utilize nitrogen efficiently, particularly in nitrogen-limiting natural systems [83,84]. BNI refers to specific chemical compounds produced and released by plant roots that can significantly suppress the activity of soil nitrifiers. Generally, plant species that have adapted to low nitrogen environments exhibit the highest BNI activity in their root systems, whereas nitrogen-fixing plants demonstrate a lower BNI capacity in the rhizosphere [85].

**Figure 2:**
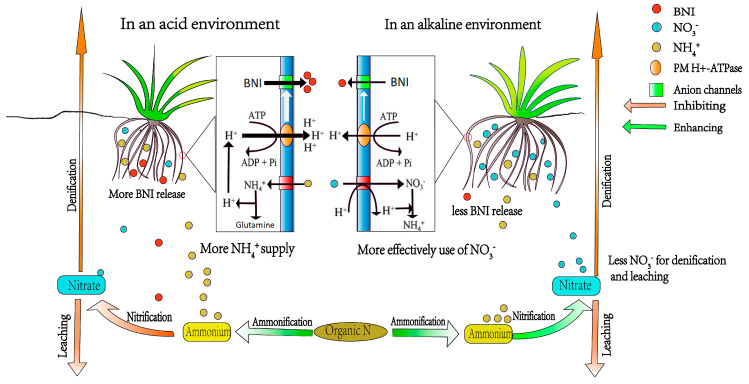
A schematic diagram illustrating how BNI release regulates exotic plant–soil N transformation, and BNI synthesis and release are highly related to plant nitrogen preference and regulated by N-form. The boxes in the center show that the hypothesis on the transport of BNIs, driven by PM H^+^-ATPase, is associated with NH_4_^+^ and NO_3_^−^ uptake and assimilation.

Previous studies have found that BNI production and release are closely related to plant nitrogen preference and are regulated by the nitrogen form (NH_4_^+^ vs. NO_3_^−^) and pH [83,86]. BNI capacity occurs only when plants utilize NH_4_^+^ as the dominant nitrogen source; however, this capacity is not observed when plants predominantly use NO_3_^−^ [50]. One study demonstrated that the presence of NH_4_^+^ or low pH around the root system alone does not trigger BNI capacity unless both factors are present simultaneously [85]. NH_4_^+^ stimulates the expression of H^+^-ATPase activity and enhances the release of BNIs from roots in the rhizosphere [87] (Figure 2). BNIs are considered anionic substances [88] and are transported through anion channels; their release is closely related to PM H^+^-ATPase activity. The latter is a universal electrogenic H^+^ pump that hydrolyzes ATP to pump H^+^ outside plant cells, generating an H^+^ electrochemical gradient that drives the process of active transport. NH_4_^+^ enhances PM H^+^-ATPase activity because its uptake depolarizes the electrical membrane potential, and the assimilation of NH_4_^+^ is a proton-generating process, leading to higher demands on H^+^ pumping activity in plant cells. This implies that root cells pump more net H^+^ under NH_4_^+^ nutrition than under NO_3_^−^ nutrition, resulting in a decrease in soil pH (Figure 2).

Although it was indicated that the optimal range for plant BNI release is pH < 6.0 in most acidic tropical grasslands or savannas [84], further research is needed to determine whether plants can release BNIs in alkaline environments with limited nitrogen supplies. Evidence suggests that the secretion of BNIs aids *L. chinensis* in surviving poor nutrient conditions and high pH [70], as BNIs can enhance NH_4_^+^ availability and reduce pH in the rhizosphere of *L. chinensis*. In contrast, the assimilation of NO_3_^−^ requires the plant to first reduce it to NH_4_^+^, a process that necessitates continuous H^+^ consumption and OH^−^ production [89] (Figure 2). Consequently, the H^+^ generated from NH_4_^+^ uptake provides the proton motive force necessary for NO_3_^−^ uptake, thereby enabling *L. chinensis* to utilize nitrogen nutrients effectively. This provides some of the first evidence that BNI may enhance nitrogen utilization efficiency in alkaline environments.

The mechanism by which invasive plants suppress nitrification through the release of bioactive nitrification inhibitors (BNIs) may contribute to their success and persistence. While BNIs can have cascading effects on other nitrogen (N) processes, such as potential changes in denitrification, and impact the structure of soil microbial communities (including the ratios of archaea, bacteria, and fungi), as well as plant biomass and primary production [90,91], there is limited information on how BNI release affects invasive plants specifically. The release of BNIs may provide a competitive advantage to exotic plants by enabling them to utilize ammonium more effectively. For instance, the invasive species *A. gayanus*, which possesses a low-affinity/high-capacity uptake system for ammonium, excels at utilizing ammonium when competing directly with native savanna plant species [29]. These native species exhibit a similar preference for nitrogen sources as *A. gayanus*, with the order of preference being NH_4_^+^ > glycine > NO_3_^−^ [92]. Additionally, microbial activity may be stimulated by BNIs released from plants, particularly in the short term, leading to increased rates of mineralization, ammonification, and nitrogen immobilization in the root zone. This is attributed to soils under high-BNI, with plants containing elevated concentrations of NH_4_^+^ and the nitrogen acquisition advantage conferred to microbes [93,94,95].

### 4.2. pH Change

Invasive plants can significantly affect soil pH, which may facilitate their own invasion as a nutrient acquisition strategy [96]. For instance, organic acids secreted by roots and rhizosphere microorganisms promote acidification, thereby increasing the solubility of nitrogen, phosphorus, and basic cations in soils [97]. Alterations in soil pH due to invasion can have a series of effects on ecosystem functions, influencing soil microbial communities, plant growth, species diversity, and plant productivity [98]. However, the relationship between the effects of invasion on soil pH and the nitrogen uptake preferences of exotic plants remains poorly understood. Some invasive plants in savannas, which exhibit a higher uptake rate of ammonium and exude compounds that inhibit biological nitrification, generally lower soil pH [29]. Other exotic plants preferring ammonium, such as *Spartina alterniflora* and *Stellera chamaejasme* L., also cause a slight decrease in soil pH [41,57]. Conversely, most exotic plants either show an increase in pH [13,99] or have no effect on it [44,65]. Because most soil organisms are particularly sensitive to changes in soil conditions, such as pH and nutrient concentration, soil pH influences the availability of different chemical forms of nitrogen (N) and N transformation.

#### 4.2.1. The Mechanism of Plant Invasions Alters Soil pH

**Figure 3:**
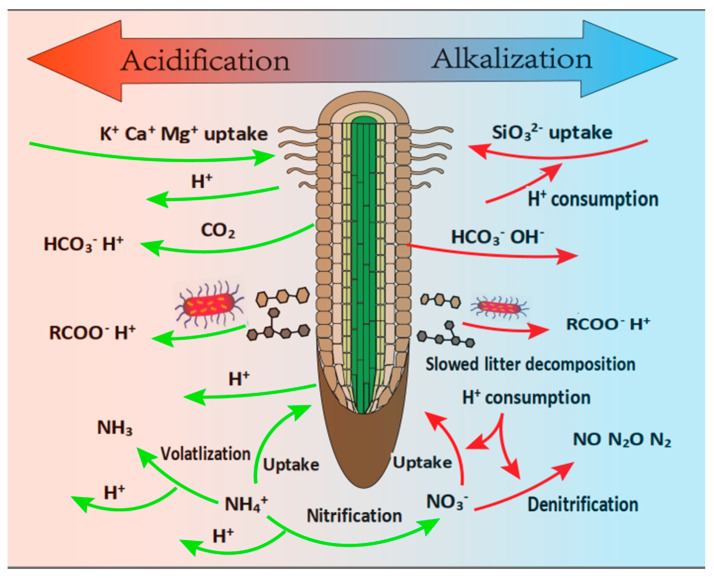
A schematic diagram illustrating that plant invasion causes soil acidification and alkalization processes in the rhizosphere. The green and red arrows represent H^+^ release and consumption, respectively. Acidification processes on the left from the top to bottom include the following: (i) cation uptake by roots coupled with the release of H^+^ ions, (ii) CO_2_ release through root exudation and respiration, (iii) organic anion release by roots to mobilize nutrients, and (iv) several N transformation processes coupled with the release of H^+^. Alkalization processes on the right from the top to bottom include the following: (i) anion uptake by roots coupled with the consumption of H^+^, (ii) HCO_3_^−^, OH^−^ release through root exudation, (iii) slowed litter decomposition reduce organic anion release, and (iv) N transformation processes coupled with the consumption of H^+^.

Plant invasions alter soil pH by shifting the balance between H^+^ production and consumption. Soil acidification is influenced by the invasive plant itself, which includes the following mechanisms (Figure 3): (i) The root uptake of nutrients in cationic form within the rhizosphere occurs through the exudation of H^+^ ions by roots to maintain cation/anion balance [100]. (ii) The generation of carbonic acid results from the dissolution of CO_2_ released by roots, microbial respiration, and the mineralization of organic matter and plant residues, all of which release H^+^ ions and consequently decrease soil pH [101]. (iii) Organic acids are produced during soil organic matter (SOM) mineralization or released by roots dissociate and release H^+^ ions, thereby acidifying the soil [101]. For example, soil pH was lower in sites invaded by the shrub *Lantana camara* compared to uninvaded sites, which is attributed to higher litter quality (high SOC, STN, and M%), faster decomposition, and increased nitrogen release [102]. Soil alkalization, also controlled by the invasive plant, includes the following processes (Figure 3): (i) The accumulation and weathering of silicates consume CO_2_ and H^+^, thus increasing soil pH [103,104]. (ii) A slowed rate of litter decomposition [105] can also increase soil pH, as the breakdown of litter produces organic acids that further elevate H^+^ concentrations [106]. (iii) Root exudates can directly increase rhizospheric pH, as the secretion of HCO_3_^−^ or OH^−^ raises rhizospheric pH to maintain charge balance [106,107].

**Figure 4:**
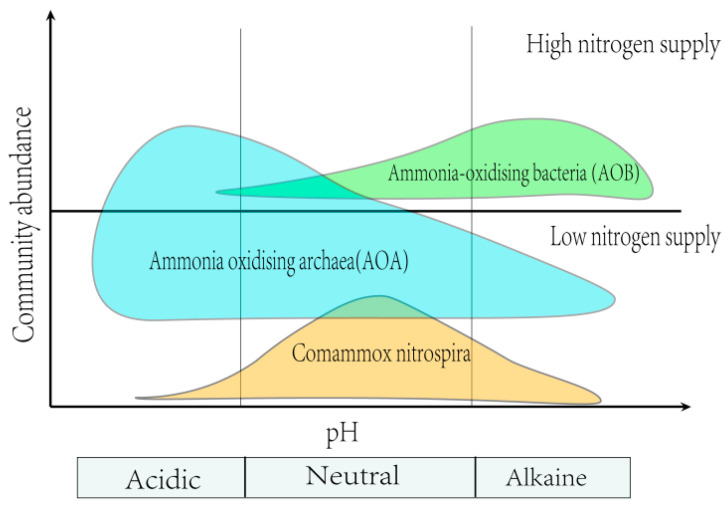
A schematic diagram illustrating that soil pH and, by extension, ammonia availability are niche-defining parameters for soil ammonia oxidizers. The different color areas represent different ammonia oxidizers’ ecological niches: ammonia-oxidizing bacteria (green), ammonia-oxidizing archaea (blue), and Comammox Nitrospira (yellow).

Plant species that differ in their ability to capture and absorb various nitrogen (N) forms, specifically their uptake preferences, can directly influence rhizosphere pH by regulating the amount of NH_4_^+^ available for nitrification [108]. It was suggested that nutrient availability largely alters soil pH indirectly, thereby structuring soil community processes [109]. Diverse N transformation processes are typically accompanied by the production and consumption of protons (H^+^) (Figure 3). For instance, the uptake of NH_4_^+^ and the volatilization of NH_3_ generate protons (H^+^), which can be offset by H^+^ consumption during mineralization, resulting in lower net H^+^ production (Figure 4). Notably, the uptake of organic nitrogen (ON) does not produce H^+^ [79]. Thus, the primary pathway leading to net H^+^ production is likely associated with nitrification [110]. A plant that preferentially takes up NH_4_^+^ would leave less NH_4_^+^ available for nitrification, thereby limiting the rate of H^+^ production from nitrogen cycling processes [25]. In contrast, plant species that favor NO_3_^−^ may release more H^+^, as a greater proportion of NH_4_^+^ is supplied for subsequent nitrification [25,111].

#### 4.2.2. Effect of pH on Soil N Transformation

Soil pH influences the availability of different chemical forms of nitrogen (N) and N transformation through several mechanisms. Mechanism (i): Soil pH is a determinant of soil organic matter (SOM) absorption on metal oxides and clay minerals [112], which in turn affects the organic and inorganic N supply for microbes. Mechanism (ii): Soil pH regulates enzymatic depolymerization, enzyme conformation, and the absorption of enzymes to clay minerals. It also influences the affinity of functional groups of enzymes and substrates [113], which can strongly regulate diverse microbial activities related to various N cycle processes. Mechanism (iii): Soil pH affects microbial community structure in relation to different N cycle processes, and individual microbial groups, particularly bacteria, have narrow pH optima for growth and activity [114].

Among all the nitrogen cycle processes, pH-mediated soil nitrification is a predominant mechanism affecting the nitrogen supply for plants, as nitrification enhances nitrogen mobility and potential loss from the soil system. A recent study suggests that soil NO_3_^−^-N levels, along with the abundances of ammonia-oxidizing archaea (*AOA*) and ammonia-oxidizing bacteria *(AOB*), increase with rising soil pH [114]. This phenomenon can be well explained by the types of ammonia-oxidizing microorganisms that thrive under varying pH conditions [115]. For instance, comammox Nitrospira prefers a neutral to slightly acidic pH and environments with low ammonia availability [116] (Figure 4). In contrast, *AOB* are more suited to neutral to alkaline environments where ammonia availability is higher [116] (Figure 4). *AOA*, on the other hand, favor low pH environments, irrespective of nitrogen application [116] (Figure 4). Since autotrophic nitrification (GAN) is predominantly controlled by AOB in non-acidic soils, higher pH levels stimulate autotrophic nitrification (GAN) [117,118]. Conversely, previous studies have reported that low soil pH creates conditions conducive to heterotrophic nitrification (GHN) [23,119]. This may be due to the fact that lowering soil pH can influence the abundance and composition of *AOA*, which are the dominant ammonia-oxidizing microorganisms in acidic soils [115]. However, a global meta-analysis indicates that there is no significant relationship between soil pH and GHN. Rather, soil total nitrogen and temperature are the primary factors driving heterotrophic nitrification, while pH primarily influences autotrophic nitrification [120].

Similarly to nitrification, the denitrification process and nitrogen (N) losses are strongly dependent on soil pH [114,121]. Soil pH can regulate the nitrification rate, which subsequently affects denitrification, as NH_4_^+^ cannot be denitrified until it is transformed into NO_3_^−^. Low pH often suppresses the growth and activity of denitrifiers [122,123], thereby limiting the magnitude of N_2_O production and resulting in lower emissions. However, N_2_O is also an intermediate product in nitrification, generated from ammoxidation and hydroxylamine oxidation. Consequently, the dominance of either processes in soil N_2_O emissions remains a topic of ongoing debate. Soil pH plays a significant role in this process, as it impacts the community structures of nitrifiers and denitrifiers, as well as enzyme activities. Recent studies have shown that the highest N_2_O emissions occur in moderately acidic soils [114]. In contrast, there is no significant linear relationship between N_2_O emissions and the quantity of N input.

Soil pH may influence the nitrogen immobilization by microorganisms, which generally prefer NH_4_^+^ as a nitrogen source [124]. However, this phenomenon does not appear to hold true for soils with lower pH levels. Research has shown that microorganisms assimilate NO_3_^−^ into organic nitrogen, contributing to 93% of the total nitrification rate in soils with pH values below 5.0 [23]. Increased rates of NO_3_^−^ assimilation into organic nitrogen can mitigate the impacts of leaching, runoff, and denitrification, effectively retaining inorganic nitrogen in acidic soil.

### 4.3. Changed Nutrient Stoichiometry Affects Exotic Plant–Soil N Transformation

Plant stoichiometry, specifically the concentrations of carbon (C), nitrogen (N), and phosphorus (P) and their ratios, provides insights into how plants adapt to environmental changes [125,126]. Generally, plants characterized by rapid growth, high specific leaf area, and elevated photosynthetic capacity [127,128] exhibit high nutrient concentrations, particularly in nitrogen and phosphorus [127]. In contrast, low nutrient plants tend to be long-lived, resilient, and possess lower metabolic capacities, which favor space occupancy and canopy dominance [129]. In terrestrial ecosystems, the C:N:P ratios of plants exhibit flexibility in response to changes in soil nutrient availability, especially when certain elements are limiting [130]. However, the overall C:N:P stoichiometry tends to remain within a narrow optimal range, a phenomenon referred to as ‘stoichiometric homeostasis.’ Mechanisms that contribute to the buffering capacity of C:N:P stoichiometry include variations in plant C:N:P ratio uptake, nutrient reabsorption [131,132], and nitrogen fixation. Nonetheless, few studies have investigated how invasive plants adjust their C:N:P ratios to achieve optimal values by altering soil nitrogen transformation processes.

Changes in nutrient stoichiometry, driven by invasive plant species, can alter the growth of soil bacteria and fungi, significantly impacting diversity–function relationships and favoring the competitive dominance of certain organisms over others. This shift may ultimately influence nitrogen transformation in the soil. As soil microbes are typically limited by carbon sources, a close relationship exists between microbial activity and soil carbon-to-nitrogen (C:N) ratios. Microbes may mineralize carbon, nitrogen, and phosphorus in proportions that reflect their optimal body C:N:P ratios [133]. Soil C:N ratios primarily affect nitrification through their influence on the abundance of ammonia-oxidizing bacteria (*AOB*) and archaea (*AOA*) [134], as well as rates of nitrogen mineralization [135]. Gross nitrogen mineralization decreases with increasing C:N ratios [136]. Soils with high C:N ratios require greater amounts of mineral nitrogen for microbial assimilation during mineralization, thereby reducing the substrate NH_4_^+^-N available for nitrifiers [137], which results in lower abundances of *AOB* and *AOA* [134] and weakens autotrophic nitrification. However, this does not hold true for heterotrophic nitrification, which relies on soil organic nitrogen content [119]. The soil carbon-to-nitrogen ratio significantly influences the rates of nitrification and denitrification [138,139]. For example, the rate of nitrification increases at lower soil carbon-to-nitrogen ratios [140], while a higher carbon-to-nitrogen ratio enhances available carbon concentrations in soils and promotes denitrification [141].

A higher carbon-to-nitrogen (C:N) ratio may be indicative of a conservative nitrogen (N) use strategy, whereas a lower C:N ratio may reflect a radical N use strategy, contingent upon the nitrogen availability in the soil. It was suggested that the invasive plant A. longifolia, which effectively utilizes both NH_4_^+^ and NO_3_^−^, accumulates litter with lower C:N ratios compared to native plants. This accumulation leads to a higher N supply and significantly increased nitrification rates in invaded soils, potentially serving as a mechanism of self-facilitation for *A. longifolia* [142]. Conversely, the invasive plant *Hieracium* can enhance carbon inputs to the soil, resulting in a higher soil C:N ratio. This change causes increased soil carbon mineralization, reduced net N mineralization, and ultimately depletes mineral N. By decreasing resource availability, *Hieracium* outcompetes other herb-field species in nitrogen-deficient tussock grasslands [143]. The stoichiometric characteristics of target plants may be altered by allelochemicals that affect the physical and chemical properties of the soil, particularly the status of nutrients [126]. A previous study [144] found that the addition of leachate from the invasive *Stellera chamaejasme* significantly stimulated soil microbial growth while decreasing phosphorus availability in the soil. Phosphorus deficiency constrained photosynthesis and CO_2_ assimilation rates, ultimately affecting plant growth and nutrient concentrations, particularly leading to a reduced C:N ratio in plants [145].

## 5. Wider Implications: Global Climate Change

Warming may increase the supply of inorganic nitrogen (N) in the soil, potentially favoring the spread of invasive plant species that possess broader soil N niches or greater flexibility in adapting to changes in preferred N forms [19]. One significant reason for the preference of different plant species for various N forms is as an adaptive strategy to cope with N limitation. Elevated temperatures enhance belowground carbon (C) allocation and root exudation, allowing plants to better explore the soil for nutrients such as N [146,147]. Increased C and N inputs subsequently boost microbial biomass and activity [101], intensifying competition between roots and microorganisms for N and phosphorus (P). This competition, in turn, enhances N mineralization and cycling [148,149], thereby increasing the availability of inorganic N for plants [149]. The production of plant roots and the input of labile C for rhizosphere organisms are particularly pronounced in nutrient-poor sites with lower total nitrogen (TN) concentrations [150,151]. However, it remains unclear whether warming could lead to progressive nitrogen limitation (PNL), which posits that the supply of N for plant growth progressively decreases in soils over longer timescales due to CO_2_ enrichment and warming, which stimulate plant growth and result in greater N sequestration in plants, litter, and soil organic matter (SOM) [152]. A crucial issue is whether N supply can meet N demand [152]. A meta-analysis [149] indicated that CO_2_ enrichment significantly accelerates the biological N fixation process, suggesting that the N demand of N_2_-fixing species may be satisfied under elevated CO_2_ conditions. However, this does not hold true for non-N_2_-fixing species. A recent study revealed that N limitations are prevalent in most natural terrestrial ecosystems, with 18% of these ecosystems experiencing significant N limitation [153].

Warming may induce species to transfer more nitrogen (N) from soil pools with typically low carbon-to-nitrogen (C/N) ratios to plant biomass pools with higher C/N ratios, resulting in an increase in soil C:N without a significant increase in total soil N. A higher soil C:N ratio may be associated with a conservative N use strategy; thus, invasive plants that effectively utilize ammonium or utilize ammonium and nitrate equally well would outcompete those plants that prefer nitrate. Furthermore, it also suggests that CO_2_ fertilization elevates the ratio of soil ammonium (NH_4_^+^) to nitrate (NO_3_^−^) [154,155], leading plants to release more hydrogen ions and acidify the rhizosphere soil [156,157].

Nitrogen (N) deposition, as discussed by Thomas [158], may alleviate progressive nitrogen limitations in response to increasing atmospheric CO_2_ levels. A global trend indicates that N deposition is expected to rise in the future [159]. Furthermore, the various forms of nitrogen components and their proportions in natural atmospheric N deposition are likely to change over time [160,161]. Notably, the rate of increase is greatest for nitrate deposition, even though ammonium remains the dominant form of nitrogen [162]. This shift may further facilitate the ability of invasive plants to effectively utilize nitrate. Additionally, nitrogen addition was shown to lower soil pH [163]; soil acidification can inhibit autotrophic nitrification (GAN). However, it may simultaneously increase the fungi-to-bacteria ratio, given fungi’s greater capacity to tolerate acidic conditions compared to bacteria [164], potentially enhancing heterotrophic nitrification (GHN) [165]. Nevertheless, this remains to be conclusively validated, as different regions and ecosystems may experience distinct nitrogen acquisition dynamics in the context of future climate change.

## 6. Recommendations for the Research Method

Although many studies have examined how nutrient preference influences plant invasion under conditions where native and invasive plants are grown separately in pots with different fertilizers [12,166,167], few studies have investigated plant nitrogen (N) preference under competitive conditions, particularly for invasive plants [168]. In some cases, nutrient preferences observed in studies of individual species often mismatch the outcomes of competition at the community level [28]. Generally, field studies manipulate interspecific interactions (competition) by removing one of the two focal species. While removal experiments can introduce unavoidable disturbance effects, they address issues related to confounding species-associated microenvironment effects, as all treatments begin with a similar composition prior to removal [169]. Additionally, these experiments mitigate concerns regarding density and plant size in competition designs [170]. Greenhouse experiments typically test competition among plants in both community-level and individual-level contexts. In community-level experiments, individuals from the target species are grown in two species combinations in larger pots compared to the smaller pots used in individual-level experiments. The community-level experiments allow us to assess whether the N form preference observed in the individual-level experiments changes in the presence of competition.

N addition treatment was applied to test whether soils with different forms and amounts of N had different effects on the physiological responses of both invasive and native plants, including survival, stem length, stem diameter, above- and belowground biomass, and estimated seed production [166]. But species’ responses to nitrogen addition are complex; sometimes the response of exotic and native species to available nutrients do not yield a general mechanism for invasion success [167].

The application of ^15^N tracer treatment can effectively elucidate preferences in the uptake of labeled N forms by various species [166] and simultaneously measure gross rates of soil N transformation. The ^15^N pool dilution method was employed to assess these gross rates, leading many researchers to develop and refine this approach, resulting in the establishment of various numerical analysis tools, such as the FLUAZ model and the N trace model family [171]. However, most studies of soil N transformation rates are carried out without plants, especially under laboratory conditions [172,173], despite the fact that soil N dynamics are potentially influenced by plant–soil interactions. Consequently, our understanding of soil N transformations may be incomplete. In addition to plant growth, the conditions under which soil is stored can also impact soil N transformations. Most studies on soil N transformation are performed on soil that was transported from the field and stored under various conditions. It remains unclear whether plants affect soil N transformations and whether different soil storage conditions significantly impact gross N transformation rates. Notably, Inselsbacher et al. [174] were the first to incorporate the effects of plants into the N trace basic model, quantifying gross rates of soil N transformations within a plant–soil ecosystem, which extended the ^15^N tracing model with a sub-model for plant N uptake including NH_4_^+^ and NO_3_^−^ uptake. Thus, in total, the N trace version used in this study consisted of six N pools and 12 processes. The results indicated that the presence of plants could stimulate N mineralization, as well as both autotrophic and heterotrophic nitrification, compared to soil alone [52]. Furthermore, N mineralization in air-dried and rewetted soil (representing different soil storage conditions) was significantly increased, whereas nitrification and immobilization decreased relative to fresh soil [52]. Thus, to realistically reflect field conditions, follow-up studies should examine gross rates of soil N transformations in the presence of plants using suitable soil–plant incubation systems with fresh soils.

In addition to plant competition, it remains unclear whether the nitrogen (N) acquisition of invasive species is driven by interspecific resource partitioning due to competition with native species or by their symbiosis with arbuscular mycorrhizal (AM) fungi. Mycorrhizal symbiosis is recognized as a significant driver of plant invasions. The association of arbuscular mycorrhizal (AM) fungi with host plants facilitates resource acquisition, thereby enhancing competitive ability [175], but the role of AM fungi in plant N uptake remains unclear [176]. Consequently, benomyl has often been applied to soils to reduce AM fungi abundance, allowing for an assessment of the role of AM fungi during invasion. Benomyl is a systemic fungicide used globally for soil fungi suppression, as it inhibits fungal cell growth during mitosis [177] but has minimal effects on plants [178]. Another approach involves categorizing plant species into non-mycorrhizal and mycorrhizal plants to reveal potential differences in acquisition patterns between these two functional plant types [179]. Hawkes et al. [62] constructed three different cylindrical in-growth cores to differentiate the effects of plant roots and AM fungi on soil N transformations: (i) a solid PVC pipe to exclude mycorrhizal hyphae and roots, (ii) a 20 μm mesh that allows hyphae to pass but not roots, and (iii) a 2 mm mesh that permits both hyphae and roots to pass.

## 7. Conclusions and Future Perspective

Exotic species optimize their nitrogen acquisition by influencing soil nitrogen trans-formations and enhancing microbial activity; this process creates a positive feedback loop that supports the fitness of exotic grasses, allowing them to maintain dominance in the community. Plants’ nitrogen uptake preferences can affect soil nitrogen transformations to optimize their nitrogen acquisition, which may depend on nitrogen availability. Generally, invasive plants can accelerate soil gross nitrogen transformations to maintain a high supply of NH_4_^+^ and NO_3_^−^ in nitrogen-rich ecosystems irrespective of their preference. However, invasive plants tend to minimize nitrogen losses to enhance nitrogen availability in nitrogen-poor ecosystems. Invasive plants with a preference for NH_4_^+^ -N are inclined to sustain high NH_4_^+^ levels by suppressing nitrification and denitrification. Conversely, exotic plants preferring NO_3_^−^-N can decrease nitrogen mineralization and autotrophic nitrification rates, directly oxidizing organic nitrogen as a primary means of producing NO_3_^−^-N in response to their nitrogen demands. Exotic plants regulate soil nitrogen (N) transformation through the release of biological nitrification inhibitors (BNIs), significantly altering soil pH and changing nutrient stoichiometry (C:N:P ratios) of soil. The synthesis and release of BNIs is closely related to the plant’s nitrogen preference and is regulated by nitrogen form (NH_4_^+^ vs. NO_3_^−^). Soil pH and variations in nutrient stoichiometry influence the plants’ availability of various chemical forms of nitrogen and affect nitrogen transformations in soil.

The interplay between the preferential nitrogen (N) uptake of invasive plant species and soil N transformation characteristics was insufficiently investigated. Understanding the combination of these processes is essential to elucidate how exotic plants optimize nitrogen use efficiency (NUE) and minimize nitrogen losses through denitrification, leaching, or runoff, which are considered critical for the success of invasive plant species. Interactions among members of the soil community must be considered, as microbe-driven alterations in N cycling may significantly impact the ecosystem’s nitrogen budget and influence the direction of ecosystem feedback related to global climate change. Further research should delve into how invasive plants make decisions regarding nitrogen acquisition, nitrogen utilization, and rhizosphere investment to enhance our understanding of alien plant invasions. This knowledge is essential for developing effective conservation strategies and for accurately predicting the consequences of biological invasions on ecosystem dynamics and responses to environmental change.

## Data Availability

All the data discussed are provided in the article.
